# A New Approach in Risk Stratification by Coronary CT Angiography

**DOI:** 10.1155/2014/278039

**Published:** 2014-09-01

**Authors:** Rine Nakanishi, Matthew J. Budoff

**Affiliations:** Los Angeles Biomedical Research Institute at Harbor-UCLA, Torrance, CA 90502, USA

## Abstract

For a decade, coronary computed tomographic angiography (CCTA) has been used as a promising noninvasive modality for the assessment of coronary artery disease (CAD) as well as cardiovascular risks. CCTA can provide more information incorporating the presence, extent, and severity of CAD; coronary plaque burden; and characteristics that highly correlate with those on invasive coronary angiography. Moreover, recent techniques of CCTA allow assessing hemodynamic significance of CAD. CCTA may be potentially used as a substitute for other invasive or noninvasive modalities. This review summarizes risk stratification by anatomical and hemodynamic information of CAD, coronary plaque characteristics, and burden observed on CCTA.

## 1. Introduction

Coronary artery disease (CAD) remains the leading cause of high morbidity and mortality in the world. Recent advanced technologies allow estimating future cardiovascular risks with correction of numerous CAD variables using several invasive or noninvasive modalities among patients with suspected CAD. These CAD variables are provided by multiple approaches such as anatomical information including the presence, extent, and severity of CAD, hemodynamic information, and coronary plaque vulnerability, which all have been widely used for risk discrimination and stratification. The recent FAME trials raised an important question regarding guiding medical management for patients who would receive benefits from revascularization based on hemodynamic or anatomical significance of CAD [1, 2]. Also, the PROSPECT study demonstrated the importance to investigate coronary plaque morphology including the severity, volume, and vulnerability provided by intravascular ultrasound (IVUS) in predicting future cardiovascular risks [3]. Since these CAD features improve the risk stratification and guide medical management, it is important to understand how to accurately select patients or examinations in a clinical setting. Another potential problem may be that most of these prospective studies were examined by invasive coronary angiography (ICA), which is the reference standard to identify the anatomical and functional significant CAD. Although diagnostic and prognostic values of ICA have been established for decades, inadequate performance of ICA can be a potential risk [4]. Thus, to avoid unnecessary ICAs, the prediction and stratification of the risk by noninvasive modalities should be considered among subjects with suspected CAD.

Coronary computed tomographic angiography (CCTA) has been widely used as a noninvasive modality for the measurement of CAD presence and severity [5–7] and risk stratification [8–10] among subjects with suspected CAD. Because of the high spatial and temporal resolution, CCTA provides anatomical information, coronary plaque burden, and coronary plaque morphology, which cannot be visualized by ICA. In addition to this benefit, a recent advanced technology of CCTA may also allow identifying hemodynamic significance of CAD. This review summarizes the utility of CCTA by the standpoints in assessing anatomical and hemodynamic variables while also detecting plaque morphology and burden for risk stratification.

## 2. Anatomical Assessment of Coronary Artery Disease by Coronary Computed Tomographic Angiography

ICA has been a gold standard to identify anatomical significance of CAD for many years. A recent large study by the American College of Cardiology National Cardiovascular Data Registry [11], however, demonstrated that nearly two-thirds of subjects undergoing ICA did not have obstructive CAD. According to the recent appropriate use criteria [12], candidates for ICA should be carefully selected and should have obstructive CAD among symptomatic patients or possibly obstructive stenosis of the left main among asymptomatic patients.

For a decade, numerous investigators have demonstrated the diagnostic utility of CCTA to identify the stenosis severity of CAD. Several multicenter studies have demonstrated the high diagnostic accuracy of CCTA [5–7]. Budoff et al. examined the diagnostic performance of CCTA compared to invasive ICA [5]. In this prospective ACCURACY study of 230 patients, CCTA demonstrated excellent diagnostic performance, particularly high sensitivity, and NPV with 95% and 99%, respectively. Because of high temporal and spatial resolutions of CCTA, CCTA has been a robust diagnostic tool to identify the presence, extent, and severity of CAD and is unlikely to miss high risk CAD.

## 3. Risk Stratification by Anatomical Assessment of Coronary Artery Disease by Coronary Computed Tomographic Angiography

Numerous previous studies demonstrated the prognostic utility of CCTA by anatomical assessment including the presence, extent, and severity of CAD [8–10]. A recent international multicenter CONFIRM study showed the anatomical stenosis severity of CAD was associated with higher mortality rate [10]. In this study of 23854 patients without known CAD, they observed that patients with nonobstructive (<50%) and obstructive CAD (≥50%) experienced 1.6- and 2.6-fold greater mortality risk compared to those with normal coronary artery. In addition to the stenosis severity alone, the extents of coronary stenosis severity in 1-vessel (HR 2.00, 95% CI 1.43–2.82, *P* < 0.0001), 2-vessel (HR 2.92, 95% CI 2.00–4.25, *P* < 0.0001), and 3-vessel diseases or left main disease (HR 3.70, 95% CI 2.58–5.29, *P* < 0.0001) were associated with greater mortality risk. These results were concordant with those of previous studies [8, 13]. Also, the mortality risk was associated with not only obstructive stenosis with ≥50%, but also nonobstructive stenosis with <50%. In 2583 subjects without known CAD and without obstructive CAD ≥50% by coronary CTA, Lin et al. demonstrated that the presence and extent of nonobstructive CAD were associated with worsening mortality during 3.1 ± 0.5 years [14]. Diffuse extended CAD is also associated with mortality. Bittencourt et al. [15] more recently explored the prognostic value of CCTA between nonobstructive and obstructive CAD and nonextensive (≤4 segments) and extensive (>4 segments) CAD. In this study of 3432 patients during a median of 3.6 years, they observed that the extensive obstructive CAD (HR 3.9, 95% CI 2.2–7.2), extensive nonobstructive CAD (HR 3.1, 95% CI 1.5–6.4), and nonextensive obstructive CAD (HR 3.0, 95% CI 1.3–6.9) were associated with the heightened cardiovascular events incorporating cardiovascular death or myocardial infarction. Of interest, subjects with extensive nonobstructive CAD experienced the greater risk compared to those with nonextensive obstructive CAD. In this regard, the presence, severity (both obstructive and nonobstructive), and extent of CAD provided by anatomical assessment are notable information for risk discrimination. With respect to guiding the decision for the medical intervention, CCTA may also be able to identify patients who would benefit from revascularization. Min et al. investigated 15223 patients without known CAD by performing CCTA and indicated that high risk CAD incorporating left main disease ≥50% stenosis, ≥3 segments with ≥70% stenosis or ≥2 segments with ≥70% stenosis, and the proximal LAD with ≥70% stenosis is associated with the benefit of revascularization compared to nonhigh risk CAD [16]. However, future randomized prospective studies examining observed variables on CCTA and its benefit for revascularization are required.

In addition to these high diagnostic and prognostic values, CCTA may play an important role as a gatekeeper to avoid inadequate performance of ICA. From the same international multicenter study, Shaw et al. [4] examined the impact of CCTA to avoid unnecessary ICA and this risk among 15207 subjects who underwent CCTA. They observed that subjects with nonobstructive CAD undergoing ICA experienced 2.2-fold greater mortality risk compared to those who did not undergo ICA. By contrast, among subjects with CAD, ICA performance reduced 39% mortality risk compared to no ICA. Hence, anatomical assessment of CAD by CCTA provides not only accurate diagnostic and prognostic implications, but also the additional notable benefit of avoiding the unnecessary ICA. Based on this concept, the ongoing prospective CONSERVE trial has been currently examined to investigate if CCTA can safely reduce unnecessary ICA as a gatekeeper.

## 4. Functional Assessment of Coronary Artery Disease by Coronary Computed Tomographic Angiography

Even though CCTA provides the high diagnostic performance, the anatomical significant coronary stenosis severity does not always predict the hemodynamic ischemia of CAD [17, 18]. Given the recent advanced technology, three methods are currently available to estimate functional significance of CAD by CCTA.

Similar to myocardial perfusion SPECT (MPS), CT perfusion (CTP) can evaluate myocardial ischemia induced by pharmacological stress. The good diagnostic accuracy of CTP has been shown by numerous previous studies, compared with MPS [19] and MRI [20]. In a recently multicenter study of CORE 320, the authors have demonstrated the high diagnostic performance of combining CCTA and CTP to identify CAD among 381 patients [21]. When compared to the traditional approach by SPECT and ICA to identify patients with hemodynamic and anatomical significant CAD, this multicenter study established that this noninvasive approach using only CCTA can similarly provide high diagnostic accuracy. A potential problem of CTP is that it requires pharmacological administration, additional radiation, and contrast medium for stress imaging since a perfusion image on both rest and stress is required for diagnosis. In the CORE 320 study previously mentioned, the mean required radiation exposure for CTP was 5.31 mSv, while that for CTA was 3.16 mSv [21].

In contrast to CTP estimating myocardial perfusion caused by the limited coronary blood flow across the stenosis on stress and rest, transluminal contrast attenuation gradient (TAG) can identify lesions-specific ischemia. TAG measures a drop in contrast medium in the coronary vessel corresponding to the coronary blood flow measured using manual or semiautomated techniques for identifying lesion-specific ischemia. The greatest advantage of this measurement is that there is no requirement for any additional contrast medium, radiation, or image acquisition compared to CTP. Several studies have examined the relation of TAG to anatomic or hemodynamic significant CAD to date. Choi et al. investigated the diagnostic accuracy of TAG compared to the stenosis severity by invasive ICA among 126 patients undergoing 64-slice CCTA [22]. They observed that increased TAG was consistently associated with stenosis severity. In addition, the combined TAG and CCTA stenosis improved classification of coronary stenosis compared to CCTA alone (area under the curve [AUC]: 0.951 versus 0.932, *P* = 0.0001), especially for calcified lesions (AUC: 0.880 versus 0.825, *P* = 0.0001). The relation of TAG to functional severity of CAD by invasive FFR was also examined by a few previous studies [23, 24]. Wong and colleagues have demonstrated that TAG provided high sensitivity, specificity, PPV, and NPV (77%, 74%, 67%, and 86%, resp.) in predicting lesion-specific ischemia by invasive FFR among 54 patients undergoing 320-slice CCTA [23]. Also, the combined TAG and CCTA improved the diagnostic accuracy in predicting functional ischemia (AUC; 0.88, 95% CI 0.81–0.96, *P* < 0.001). Another study by Yoon et al. explored the diagnostic accuracy of TAG and FFR_CT_ compared to invasive FFR among 53 patients undergoing 64-slice CCTA [24]. In this study, TAG demonstrated a modest association with invasive FFR (*γ* = 0.379, *P* < 0.001) with a sensitivity, specificity, PPV, and NPV of 38%, 88%, 67%, 69%, respectively. A potential issue is that TAG only assesses a contrast attenuation cooperating in the delay of coronary flow across the stenotic lesion in a coronary vessel in the rest condition. Based on these limited numbers of previous studies, the severity of TAG is likely to be associated with anatomical stenosis severity of CAD but may not relate well to functional ischemia.

A current innovative technology allows assessing fractional flow reserve (FFR) to identify the lesion-specific ischemia by CCTA (FFR_CT_). FFR_CT_ is a novel method provided by an advanced technique to identify lesion-specific ischemia by invasive FFR and is computed from various anatomic and physiologic pieces of information as well as fluid dynamics observed on CCTA [25]. To date, 3 prospective studies investigating the diagnostic performance of FFR_CT_ were examined. DISCOVER-FLOW is the first multicenter prospective study examining the diagnostic accuracy of FFR_CT_ in identifying lesion-specific ischemia by invasive FFR among 103 patients undergoing CCTA and ICA with FFR [26]. Second, the larger multicenter DeFACTO study examined the diagnostic accuracy among 252 patients with stable CAD [27]. Both studies showed 73–87% accuracy, 90–92.6% sensitivity, and 84–90.4% NPV. Sequent to these studies, the most recent multicenter NXT study of 254 patients [28] showed an AUC for FFR_CT_ with greater diagnostic performance compared to that of CTA stenosis alone (0.9 versus 0.81, *P* = 0.0008, resp.). The greatest benefit of this novel method is that it requires no additional contrast, radiation, image acquisition, or pharmacological stress but provides high accuracy using only rest CCTA images in identifying lesion-specific ischemia. Also, compared to TAG, FFR_CT_ is not likely to be affected by the number of cardiac cycles needed for acquisition. The potential issue of this technique might be that it requires 6 hours to calculate FFR_CT_. However, the time requirement for assessing FFR_CT_ has been lately improved using the latest-generation computational fluid dynamic techniques.

As mentioned previously, CCTA could be a “gatekeeper” to avoid unnecessary ICA performance. On the other hand, CCTA paradoxically may raise the incidence of ICA performed compared to other noninvasive modalities. A prospective SPARC study by Hachamovitch et al. [29] investigated the frequency of ICA or revascularization after noninvasive tests including CCTA, SPECT, or PET. They observed that patients with abnormal findings after each noninvasive test were more likely to undergo ICA compared to those without abnormal findings. Even after normal/nonobstructive and mild test findings, however, the finding of CCTA stenosis resulted in the higher frequency of ICA compared to SPECT or PET. This potential paradoxical issue may be caused by the lower specificity of CCTA in identifying anatomical stenosis severity because of the overestimation of stenosis by visual assessment. With respect to this issue, a combination of both anatomical assessment and hemodynamic assessment of CAD may be a more robust “gatekeeper” by increasing the incremental diagnostic accuracy while maintaining higher sensitivity and specificity compared to anatomical assessment alone.

## 5. Risk Stratification by Functional Assessment of Coronary Artery Disease by Coronary Computed Tomographic Angiography

Noninvasive hemodynamic assessment such as myocardial SPECT imaging has been universally considered as a robust approach for evaluation of the presence and severity of hemodynamic significant CAD, the future cardiovascular risk, as well as decision-making for the optimal medical intervention or revascularization [30, 31]. A multicenter FAME study reported by Tonino et al. [1] demonstrated that functional assessment of CAD by invasive FFR was superior to anatomical assessment by ICA for risk stratification among patients with stable CAD. In this study, they guided decision-making for revisualization based on functional assessment which improved the MACE risk compared to the anatomical assessment. As noted earlier, three methods were utilized to identify hemodynamic significant CAD on CCTA; however, the utility for risk stratification by these methods has not yet been investigated to date. The benefit of CCTA is an allowance to assess functional significance of CAD followed by anatomical information using only one modality. This could save time and costs and reduce the potential risk by inadequate performance of ICA and/or invasive FFR to clarify evidence of significant ischemia. The utility of hemodynamic assessment by CCTA for risk stratification and the decision-making for the optimal medical intervention warrant further investigation.

## 6. Assessment of Coronary Plaque Type and Vulnerability by Coronary Computed Tomographic Angiography

Since ICA did not display high grade stenosis of coronary artery with ≥70% among 50% or more patients having myocardial infarction [32], ICA without obstructive CAD does not always imply an excellent prognostic value. The potential mechanism underlying why ICA misses the coronary lesion associated with future cardiovascular events may be because ICA cannot show the visualization of coronary plaque type and/or vulnerability associated with plaque rupture beyond stenosis. The coronary plaque with vulnerability is the so-called vulnerable plaque [33]. In previous pathology studies, this adverse plaque is more likely to contain large necrotic cores, a thin-cap fibroatheroma (TCFA), positive arterial remodeling, and/or spotty calcification, in which all of compositions are associated with cardiovascular events [33, 34]. Previous studies demonstrated that the adverse plaque can be visualized using invasive modalities such as IVUS and cardiovascular optical coherence tomography (OCT), and the adverse plaque assessed by these modalities provides a good correlation with that observed on CCTA [35–37]. The findings of the vulnerable plaque on CCTA are represented by low attenuation plaque (LAP), positive remodeling, or spotty calcification [38].

Clinical characteristics are essential factors; however, it is not always accurate to estimate the future cardiovascular risk. A recent investigation by Bourantas et al. [39] examined the relationship of clinical and angiographic characteristics to high risk plaque, defined as plaque with ≥2 high risk features including TCFA, plaque burden ≥70%, and/or minimal luminal area ≤4 mm^2^ detected by IVUS. Compared to subjects with low risk plaques, they observed that those with high risk plaques experienced higher cardiovascular risk (Hazard ratio [HR] 2.63, 95% CI 1.62–4.26, *P* < 0.001). Also, high risk patients possessed a higher Framingham risk score and a greater extent of CAD by ICA. Nevertheless, baseline clinical and angiographic characteristics had an only modest impact to predict high risk patients (AUC for clinical characteristics versus clinical and angiographic characteristics; 0.55 versus 0.64). Since the conventional CAD risk factors do not always identify vulnerable plaque, the visualization of the plaque type or vulnerability on CCTA by modalities should be required.

## 7. Risk Stratification by Coronary Plaque Type and Vulnerability

Whereas numerous studies demonstrated that vulnerable plaque detected by IVUS/OCT is likely to be observed among patients with acute coronary syndrome compared to those with stable angina [40–43], these invasive methods cannot routinely be allowed to stratify the risk among subjects with suspected CAD.

There were only a few studies showing the relation of plaque futures on coronary CT to cardiovascular events to date [44, 45]. Our group previously examined 1102 symptomatic subjects with nonobstructive coronary artery disease detected by electron-beam CT and demonstrated that nonobstructive noncalcified plaque (NCP) or partially calcified plaque (PCP) inclusive of both noncalcified and calcified plaque components, as compared to calcified plaque (CP) alone, imparted a higher prevalence of long-term adverse clinical outcomes in a follow-up of 78 ± 12 months [44]. In this study, subjects with NCP experienced 7-fold higher incident mortality compared to those with CP (9.6% versus 1.4%) and 3-fold higher than those with PCP (9.6% versus 3.3%). However, the finding included a potential issue with limited assessment of plaque type by electron-beam CT. Noncalcified plaque components detected by CCTA include all fibrous, fibro fatty, or necrotic cores, all of which provide different risks of future cardiovascular events [46]. Necrotic cores are more likely to relate to plaque rupture [33].

Limited studies have shown that the adverse plaque on CCTA was associated with future cardiovascular events [47, 48]. Motoyama et al. examined the coronary plaque features between 38 acute coronary syndrome (ACS) and 33 stable angina pectoris (SAP) patients who underwent CCTA before percutaneous coronary intervention. In the ACS group, plaque characteristics with positive remodeling, LAP with <30 HU, or spotty calcification were highly observed compared to SAP group. Subsequently, they prospectively investigated the relation of these adverse plaque features to the future ACS risk among 1059 patients undergoing CCTA [48] and have demonstrated that subjects with adverse plaque including both positive remodeling and LAP possessed almost 23-fold higher risk of future ACS compared to those without any adverse plaque feature or any plaque. In this regard, the vulnerability of coronary plaque may be an additional essential aspect to predict future cardiovascular events. While being prognostically useful, the utility of aggressive medical intervention such as coronary revascularization for these plaque features remains yet unexamined.

## 8. Risk Stratification for Asymptomatic Patients

Traditional clinical risk assessment, such as Framingham risk scores (FRS), has been used universally to stratify the future 10-year cardiovascular risk among asymptomatic patients. However, FRS may underestimate the risk. By contrast, coronary artery calcium (CAC) scanning is a diagnostic tool to determine the presence and extent of calcified plaque in coronary arteries by noncontrast CT and an examination for early detection of CAD and improvement of risk stratification for individuals at low-intermediate or intermediate CAD risk. A prospective population based study by Greenland and colleagues [49] explored the relation of FRS and CAC score (CACS) to future nonfatal MI or cardiac death among 1461 asymptomatic individuals during a median of 7 years of follow-up. They observed that the FRS + CACS predicted future nonfatal MI or cardiac death compared to FRS alone (AUC; 0.68 versus 0.63, *P* < 0.001). A similar result was reported by another population based MESA study. Yeboah et al. [50] demonstrated that CACS provided a greater prognostic value of future cardiac events incorporating myocardial infarction, angina followed by revascularization, cardiac arrest, or CHD death compared to FRS alone or FRS and any other clinical features such as ankle-brachial index, high-sensitivity CRP, or family history among asymptomatic individuals with intermediate risk.

Current guidelines do not recommend CACS screening among asymptomatic patients with low or low-intermediate risk due to the low absolute event rates [51–53]. However, several studies demonstrated the high prevalence of CACS as well as the prognostic impact of CACS among low risk patients. Nasir et al. explored that increased CAC predicts higher mortality risk among asymptomatic subjects with lower number of CAD risk factors [54]. Similar results in predicting CHD risk were reported by Silverman et al. [55]. Also, these studies demonstrated the high prevalence of CACS, showing that 30–50% of patients had some CACS among subjects with CACS zero. In this regard, traditional risk factors may underestimate the prevalence of coronary atherosclerotic burden and risk. These previous findings suggested that CACS is a robust “visualized CAD risk factor” and its impact is over that of traditional risk factors for identification, discrimination, and stratification of the risk.

Numerous previous studies investigated the utility of CACS in predicting all-cause death or cardiovascular events [56–60]. Budoff et al. investigated the long-term prognostic value of CACS at a mean follow-up of 7.6 years among 25253 asymptomatic patients and found that CACS provides incremental information in addition to the total number of clinical risk factors and age [56]. Also, the 12-year survival rate in patients with CACS zero is extremely high with 99.4%. The MESA study has recently collected long-term follow-up data and will investigate the utility of CACS for not only mortality but also coronary heart disease (CHD) or coronary vascular disease (CVD) risks. CACS is calculated by the sum of coronary calcified plaque lesions with density greater than or equal to 130 Hounsfield units (HU) having an area ≥1 mm^2^. This lesion score is simply measured by the maximal CT numbers, providing the total CACS by the corrections of all lesion scores that is associated with the risk. However, it has not been yet examined whether the volume metric and morphology of CACS are associated with the cardiovascular risk. More recently, the MESA group explored that the increased CAC volume is also associated with CHD or CVD. Additionally, density of CACS is inversely associated with these risks [61]. These new CAC features may be another important potential metric of CACS for risk reclassification among asymptomatic patients.

The utility of CCTA to improve the risk stratification over CACS among asymptomatic subjects is still controversial. A large multicenter study examined whether CACS was predictive of future cardiac events compared to CCTA. In the study of 7590 asymptomatic patients without known CAD, Cho et al. [62] observed any CCTA findings incorporating Duke coronary artery disease prognostic index, segment stenosis score, or segment involvement score as the severity, extent, and location of CAD by CCTA did not impact on the incremental prognostic value over models with CACS and FRS. With respect to these results, the assessment of only subclinical atherosclerotic burden such as CACS has had a clinically robust prognostic impact among asymptomatic patients compared to any other data observed on CCTA, but that did not incorporate plaque type or vulnerable plaque in this study. In this regard, the current guideline does not recommend performing CCTA as a screening purpose among those subjects presenting no chest symptoms [30]. When patients possess high clinical CAD risk, however, CCTA may be considered to be performed for risk assessment. Min et al. [63] recently investigated the impact of CCTA for risk discrimination among 400 asymptomatic patients with diabetes, demonstrating the extent and severity of CAD by CCTA provided the incremental value to predict MACE over CACS. The subclinical coronary atherosclerotic burden determined by CACS provides the independent and incremental prognostic utility in addition to traditional CAD risk factors among asymptomatic subjects; however, the variables observed on CCTA such as stenosis severity or extent of CAD may provide the incremental prognostic value over CACS alone among those with high risk such as diabetes. In addition, even when patients had no chest symptom or atypical chest pain, the previous study demonstrated that 4.3% or 15.5% of patients with intermediate or high FRS had vulnerable plaque on CCTA, while those with low FRS did not have any vulnerable plaque [64]. Although no studies have explored the prognostic impact of vulnerable plaque on CCTA among asymptomatic population to date, CCTA may have potential to identify CAD associated with future cardiac events among those with high FRS risk.

## 9. Risk Stratification by Coronary Atherosclerotic Burden for Symptomatic Patients

The prognostic impact of coronary atherosclerotic burden defined as CACS for symptomatic patients has been also reported. In the study examining the relation of CACS and CCTA findings to MACE including cardiac death, nonfatal myocardial infarction, or coronary revascularization among 4425 symptomatic subjects reported by Hou and colleagues, the combined CACS and CCTA findings with stenosis severity provided the incremental prognostic information in predicting MACE over the combination of risk factors and CACS or the risk factors alone (AUC: 0.92 versus 0.82 versus 0.68) [65].

Although the coronary atherosclerotic burden as determined by CACS provides the prognostic value among either asymptomatic or symptomatic subjects, the presence, severity, and extent of noncalcified plaque are not visualized on CAC scanning, but that can be identified by CCTA. In a previous publication by Kristensen and colleagues [45] who investigated nonculprit nonobstructive coronary plaques on CCTA in 312 consecutive patients presenting with non-ST-segment elevation myocardial infarction, nonobstructive NCP volume was independently and closely associated with an increase in intermediate-term 16-month cardiac events (nonobstructive NCP volume (per 100 mm^3^ increase): HR 1.18, 95% CI 1.06–1.31, *P* = 0.002). The volume of nonculprit and noncalcified plaque may be associated with cardiovascular events. Although the prognostic impact links to not only calcified plaque volume, but also noncalcified plaque volume, the metric assessment of plaque volume may be time consuming. Recently, novel techniques can allow the measurement of coronary plaque volume observed on CCTA including calcified and/or noncalcified plaque using semiautomated CT software [66, 67], with high accuracy compared to IVUS [68]. The relation of the total coronary plaque volume as determined by the overall coronary atherosclerotic burden in predicting the future cardiovascular events has not yet been examined. Further studies could be helpful to determine whether the overall coronary atherosclerotic burden observed on CCTA provides incremental prognostic value in addition to other CCTA information.

## 10. Risk Stratification by Plaque Progression

The one-point assessment of coronary atherosclerotic burden may be limited in predicting future cardiac events since the presence of coronary plaque accelerates the plaque progression as well as the risk. At present, the prognostic utility of coronary plaque progression assessed by CT has been investigated by several studies [69, 70]. Budoff et al. have explored the prognostic impact of CACS progression among 4609 asymptomatic patients who underwent serial noncontrast cardiac CT [69]. In this study, they observed that the progression of CACS was significantly associated with worsening mortality. Subsequently, they also examined the relation of CACS progression to future CHD among 5682 asymptomatic patients with serial scans from the large multicenter MESA study with longer follow-up of a median of 7.6 years and similar results were observed [70]. In these studies, subjects with positive CACS are likely to experience the increased mortality or cardiovascular risk. The serial measurements of CACS may be beneficial, in particular among patients with high risk such as high baseline CACS.

By contrast, the utility of serial assessment of plaque progression by CCTA has not yet been investigated. A limited study by Lehman et al. examined the utility of serial CCTAs in assessing the progression of coronary plaque [66]. In this study of 69 patients with acute chest pain, the coronary plaque burden measured by semiautomated CT software significantly increased in 2 years and that was associated with clinical risk factors. With respect to the serial CT studies using either noncontrast CT or contrast CCTA, the additional radiation dose may be a potential issue. However, the required radiation dose for noncontrast CT is approximately <1–3 mSv [71, 72], which is relatively low. Also, the recent technology allows decreasing radiation dose for CCTA by the reduction of tube voltage and/or tube current [72]. In particular, current high pitch coronary CTA models may be able to provide radiation dosed within <0.5 mSv for CAC scanning [73] and <1 mSv for CCTA [74].

Since the serial assessments by noncontrast cardiac CT can only provide the development of calcified plaque burden, it would be of great interest to investigate whether the overtime change of coronary plaque severity, type, vulnerability and total plaque volume by serial CCTAs is associated with future cardiovascular risk.

## 11. The Future Potential Approach to Stratify Cardiovascular Risk by CCTA

CCTA has been a robust noninvasive modality to estimate not only the presence, extent, and severity of CAD, but also hemodynamic significance of CAD as well as coronary atherosclerotic plaque characteristics or burden. For a higher diagnostic performance of CCTA, the combined approach by both anatomical and hemodynamic assessments might be useful, since this approach improves false positive rates provided by the anatomical assessment alone because of the overestimation of coronary stenosis. While being diagnostically useful, a potential for risk stratification by the combined assessment is still unclear.

Several previous studies demonstrated the incremental prognostic value of anatomic and hemodynamic assessment of CAD by CCTA and SPECT to date [75, 76]. Pazhenkottil and colleagues examined the prognostic value of hybrid CCTA/SPECT study among 302 subjects with suspected CAD [75]. In this study, the patients with abnormal perfusion SPECT and CAD with ≥50% or those with either abnormality experienced higher MACE (all-cause death, nonfatal MI, unstable angina requiring hospitalization, and coronary revascularization), or death, or myocardial infarction when compared to those without any abnormality. Another study by Kawai et al. examined the utility of the combined assessment with CCTA and SPECT among 204 patients who underwent coronary artery bypass grafting [76]. They observed the combination between unprotected coronary territories defined by the number of significant stenoses with left main ≥50%, other native coronary artery stenoses ≥70%, or graft stenosis ≥70% by CCTA and summed stress score by SPECT improved the prediction of cardiovascular events incorporating cardiac death, nonfatal myocardial infarction, unstable angina requiring revascularization, and heart failure hospitalization. The combination of anatomical assessment and hemodynamic assessment of CAD could provide incremental prognostic value.

There were only a few studies demonstrating the combined evaluation of coronary plaque characteristics and other CAD features to predict cardiovascular risk [3, 9]. In the PROSPECT study by Stone et al. [3] who investigated the natural history of coronary plaque by IVUS and the risk among 697 patients with acute coronary syndromes who underwent ICA, they observed the presence of ≥70% stenosis, TCFA and minimum luminal area <4 mm^2^ showed 5.03, 3.35, and 3.21-fold increased future major adverse cardiovascular events (MACE). When combining all features, the MACE risk increased 11-fold higher compared to that of CAD without these plaque features. The finding suggested that all plaque characteristics including the severity, burden, and vulnerability of coronary plaque were associated with MACE risk and this combination predicted the greatest MACE risk. Another study by van Werkhoven et al. [9] has investigated the prognostic value of CCTA and SPECT among 541 subjects with suspected CAD. They observed that the combined clinical characteristics, abnormal myocardial perfusion by SPECT, stenosis severity ≥50% by CCTA, and the presence of noncalcified plaque by CCTA showed the greatest incremental prognostic value when compared to clinical characteristics alone, clinical + stenosis severity ≥50%, clinical + abnormal perfusion, or clinical + abnormal perfusion + stenosis severity ≥50%, a finding that suggested the combination of anatomical, hemodynamic significant CAD and plaque type may be a greater potential for risk stratification.

Since cardiac death or myocardial infarction occurs because of multifactorial interaction, the single approach may be limited to identify CAD associated with the future cardiac events. It would be of great interest to take into account all available information such as anatomical and hemodynamic significance of CAD as well as coronary atherosclerosis incorporating the burden, type, and vulnerability for the risk stratification and the guide in the medical management among subjects with suspected CAD. However, these variables may be excessive information for patients with low CVD risk. The appropriate selection in identifying individuals who are at high risk of future cardiac events should be carefully considered based on chest symptoms (asymptomatic versus symptomatic), CAD risk factors including FRS, or the history of known/unknown CAD. Although CACS has been a robust prognostic tool among asymptomatic patients, the utility of CACS may be extended to those with a low risk. With respect to those with a high risk of CAD such as diabetes or high FRS, CCTA may be a potential for risk stratification. By contrast, more detailed information regarding CAD observed on CCTA would be needed for risk stratification among patients with high risk of CAD such as symptomatic, high FRS, or history of known CAD. Given the novel method to assess functional significance of CAD by CCTA, the relation of this new approach to CVD risk has not been examined to date. However, the hybrid assessment by CCTA including anatomical and hemodynamic information, coronary plaque characteristics, and atherosclerotic burden may have a potential role in stratifying CVD risk.

## 12. Conclusion

CCTA is the only robust noninvasive modality to evaluate the presence and extent of CAD, anatomical and hemodynamic severity of CAD, coronary plaque characteristics—type and vulnerability—and the atherosclerotic burden. With taking advantage of these CCTA abilities, all available data of CAD observed on CCTA may improve identification, discrimination, and reclassification of the future cardiovascular risk.
